# Vertical transmission of Zika virus and the repercussions on children: the knowledge is not complete

**DOI:** 10.1186/s12916-021-01901-0

**Published:** 2021-02-05

**Authors:** M. I. Moreira, S. H. Villela, M. D. B. B. Meio

**Affiliations:** Instituto Fernandes Figueira/Fiocruz, Avenida Rui Barbosa, Rio de Janeiro, 716 Brazil

**Keywords:** Zika virus, Neurodevelopment, Children

## Background

The association between Zika virus (ZIKV) infection in pregnancy with neurological abnormalities such as microcephaly, brain calcifications, epilepsy, eye and hearing impairments, and developmental delays has been described in recent years [1–3]. Despite several published studies reporting development delays in children after prenatal exposure to ZIKV, several doubts remain and hinder ongoing studies, mainly related to the absence of serological tests capable of identifying asymptomatic cases, making it difficult to implement unexposed groups in the cohorts [1–4]. This is further complicated by the difficulty of diagnosing ZIKV infection retrospectively, because of the narrow period of viremia and short-lived immunoglobulin M (IgM) responses, plus serologic cross-reactivity with dengue virus, highly prevalent in many countries [4, 5].

### Child neurodevelopment and Zika virus exposure

Several studies in different countries have found a worse neurodevelopment score in children exposed to ZIKV who were born apparently normal [2, 3, 6]. However, the major criticism of these studies is the absence of a control group, including children provenly known as not being exposed to ZIKV during pregnancy. A cohort study from Brazil and Colombia with assessment at different ages and different neurodevelopment tests demonstrated that children exposed to ZIKV in utero are at risk for a spectrum of functional neurologic and developmental adverse outcomes, especially in the language domain. These dysfunctions occur in children born without microcephaly, but these studies did not have a control group. The most important issue about the lack of a control group is the limitation of serological ZIKV laboratory testing. In an endemic area, there are many asymptomatic infected women and, unfortunately, it is almost impossible to detect all of them. Serological tests on children are also a challenge. Children serologic neutralizing antibody positivity before 18 months of age may reflect both maternal antibody transfer and postnatal acquisition [3, 6].

Another challenging point for cohort studies in children is choosing a neurodevelopmental test for different ages that is culturally validated and translatable to the native language. Various factors can affect the results, such as the parent’s mental health, the school level, the economic situation, the child’s potential health problems in the first year of life, breastfeeding policies, prematurity, and others. Several different tests had been used in ZIKV cohort studies such as Bayley III tests, ASQ, Warner Initial Developmental Evaluation of Adaptive and Functional Skills (WIDEA), and the Alberta Infant Motor Scale (AIMS), among others [3, 6, 7]. However, independent of the test used, the influence of ZIKV exposure on children’s neurodevelopment is consistent [3, 6]. Another study in Brazil, which included children before the ZIKV epidemic occurred, showed that less than 10% of alterations in any domain of the Bayley III test were found in children that were born at term [8].

In this volume of *BMC Medicine*, Grant et al. published a prospective, well-written, study of the outcome of children, prenatally exposed to ZIKV, at 24 months of age. It is a population-based mother-child cohort study of women whose pregnancies overlapped with the 2016 ZIKV epidemic in Guadeloupe, Martinique, and French Guiana. Their inclusion criteria were well determined to define ZIKV exposure during pregnancy. Infants were included in this analysis if maternal ZIKV infection during pregnancy could be determined. Other factors that can influence child neurodevelopment have also been considered as inclusion factors: gestational age, abnormal transfontanelle cerebral ultrasound findings after delivery or no abnormal ultrasound findings on the last ultrasound performed during the third trimester of the mother’s pregnancy, and absence of microcephaly at birth. Neurodevelopment assessments involved the Ages and Stages Questionnaire (ASQ) for five dimensions of general development—communication, gross motor, fine motor, problem-solving, and personal-social skills. They included 156 toddlers with and 79 toddlers without in utero ZIKV exposure who completed their neurodevelopment assessments. Their results were surprising because they found minimal apparent differences in neurodevelopment outcomes compared to ZIKV-unexposed toddlers at 24 months of age. However, there was a great loss of children in the control group, and this might have included a selection bias in the results. They performed other statistical analysis to decrease these biases and presented a good discussion about that [7].

Nevertheless, recommendations for longitudinal infant and child follow-up would be appropriate in children born during the ZIKV epidemic. These children are reaching school age, and to follow them at this age could be especially important in the setting of symptomatic and asymptomatic maternal infection, until a better understanding of this congenital disease could be reached. At an early age, there is a window of opportunity in which if some delays are found, and adequate interventions could be applied in order to improve their learning opportunities and quality of life.

However, the final answer to several issues addressed here will probably come from the ZIP Study from National Institute of Health (NIH) [9]. In the NIH study, pregnant women were included in the first trimester of pregnancy and followed prospectively with serological tests (Zika Mac Elisa from CDC) and RT-PCR in the blood monthly and RT-PCR in the urine every 15 days. All children are being followed from birth to the end of the first year of life. Another important study that is going on is the systematic review and metanalysis conducted by WHO. The principal investigators (PIs) and international leaders in ZIKV research have formed the ZIKV Individual Participant Data (IPD) Consortium to identify, collect and synthesise IPD from longitudinal studies of pregnant women that measure ZIKV infection during pregnancy and fetal, infant or child outcomes [10]. Results from both studies are not yet available, but important responses are expected to arrive shortly with the publication of these results.

## Conclusion

In conclusion, there is still knowledge to be built on the repercussions of vertical transmission of ZIKV and its consequences on the child’s and family lives. The epidemic is over, but probably, there are still isolated cases happening. Children are reaching school age and still need to be followed up. Currently, mothers live with their children outside health care facilities, daycare centers, and places for adequate essential stimulation and interventions, with reduced care on account of the COVID-19 pandemic. The opportunities that were few are now even smaller for all exposed children and for those born during the period of the ZIKV epidemic.
